# Olive leaf extract prevents obesity, cognitive decline, and depression and improves exercise capacity in mice

**DOI:** 10.1038/s41598-021-90589-6

**Published:** 2021-06-14

**Authors:** Toshio Mikami, Jimmy Kim, Jonghyuk Park, Hyowon Lee, Pongson Yaicharoen, Sofya Suidasari, Miki Yokozawa, Ken Yamauchi

**Affiliations:** 1 Department of Health and Sports Science, Nippon Medical School, Tokyo, 180-0023 Japan; 2 Department of Pharmacology, Graduate School of Medicine, Nippon Medical School, Tokyo, 113-8602 Japan; 3 Department of Physiology, Chiang Mai University, Chiang Mai, Thailand; 4 Nutrition Act. Co. Ltd., Tokyo, 104-0061 Japan

**Keywords:** Neurophysiology, Mitochondria, Fat metabolism

## Abstract

Obesity is a risk factor for development of metabolic diseases and cognitive decline; therefore, obesity prevention is of paramount importance. Neuronal mitochondrial dysfunction induced by oxidative stress is an important mechanism underlying cognitive decline. Olive leaf extract contains large amounts of oleanolic acid, a transmembrane G protein-coupled receptor 5 (TGR5) agonist, and oleuropein, an antioxidant. Activation of TGR5 results in enhanced mitochondrial biogenesis, which suggests that olive leaf extract may help prevent cognitive decline through its mitochondrial and antioxidant effects. Therefore, we investigated olive leaf extract’s effects on obesity, cognitive decline, depression, and endurance exercise capacity in a mouse model. In physically inactive mice fed a high-fat diet, olive leaf extract administration suppressed increases in fat mass and body weight and prevented cognitive declines, specifically decreased working memory and depressive behaviors. Additionally, olive leaf extract increased endurance exercise capacity under atmospheric and hypoxic conditions. Our study suggests that these promising effects may be related to oleanolic acid’s improvement of mitochondrial function and oleuropein’s increase of antioxidant capacity.

## Introduction

Since obesity is a risk factor for development of metabolic disorders and cognitive decline^1–3^, prevention and treatment of obesity is essential for health. Dietary control and regular exercise have both been shown to be effective strategies for obesity prevention, with many studies reporting that regular exercise is also effective at maintaining cognitive function^4–6^. There are many people, however, who cannot participate in regular exercise regimens for various reasons, including time constraints and physical limitations. Therefore, it is important to identify other methods to achieve similar, or even enhanced, health effects as exercise. Olive leaf extract (Ole) may have these desired effects.

While the fruit of the olive plant (*Olea europaea*) is commonly used to produce cooking oils, the leaves have been used as a traditional remedy to treat inflammation, ulcers, and tumors for many years^7^. Olive leaf extract contains many polyphenols, including oleuropein, a compound that has been shown to lower inflammation, blood pressure, and cholesterol levels^8^. Olive leaf extract also contains oleanolic acid, a potent agonist of transmembrane G protein-coupled receptor 5 (TGR5)^9^. TGR5, a widely present receptor in the body, is activated by bile acids and leads to increased intracellular cyclic adenosine monophosphate (cAMP) levels and energy production in cells, resulting in decreased fat tissue and weight^10^. Oleanolic acid also has been reported to prevent obesity in mice fed a high-fat diet (HFD)^11^. Olive leaf extract has been shown to contain 5% or more of both oleanolic acid and oleuropein, with proven anti-inflammatory effects induced by suppressed secretion of inflammatory cytokines^12^ and with anti-obesity effects identified in mice fed an HFD^13^.

Activation of TGR5 by agonists like oleanolic acid also increases peroxisome proliferator-activated receptor gamma coactivator 1α (*PGC-1α*) and Sirtuin1 (*Sirt1*) expression and enhances mitochondrial biogenesis^14^. Mitochondrial dysfunction, particularly defective mitochondrial biogenesis, is an early and prominent feature of many neurological disorders, including Alzheimer’s disease^15^. Neuronal mitochondrial dysfunction is one of the main factors contributing to the cognitive decline observed in these disorders^16^, with the restoration of mitochondrial function demonstrated to recover cognition^17^. These previous studies led to our hypothesis that administration of olive leaf extract, with its large amount of oleanolic acid, would not only prevent obesity, but would also help to maintain cognition through increased cerebral mitochondrial biogenesis induced by TGR5 activation.

Oleuropein, another significant component of olive leaf extract, has been shown to have potent antioxidant effects in vivo and in vitro and is known to lend a bitter pungency to extra-virgin olive oil^8^. High caloric intake and lack of exercise, both characteristics of a typical modern lifestyle, can contribute to development of obesity, as well as to increased oxidative stress and accumulation of oxidative products in the brain^18^. Olive leaf extract, with its large amount of oleuropein, is believed to scavenge reactive oxygen species in the brain and to thereby help maintain cognitive function.

In skeletal muscle, regular exercise has been shown to increase expression of PGC-1α and subsequent mitochondrial biogenesis, leading to improved endurance exercise capacity. A previous study showed that increased levels of PGC-1α in skeletal muscle were essential for improving endurance exercise capacity^19^, with expression levels dependent on the intensity of exercise^20^. Overexpression of PGC-1α-b has also been shown to promote mitochondrial biogenesis and increase fatty acid transport expression, resulting in enhanced exercise capacity^21^. Moreover, overexpression of PGC-1α in skeletal muscle has been shown to increase muscular hypertrophy ^22^. These previous studies suggest that intake of olive leaf extract may increase mitochondrial biogenesis and improve exercise capacity.

Although high caloric intake and lack of exercise are typical features of a modern lifestyle and both are risk factors for various diseases, many more studies have reported the adverse effects of high caloric intake than that of inactivity. Worldwide, the strongest highest risk factor for death is hypertension, followed by smoking, diabetes, and inactivity (lack of exercise)^23^. In the human study, physical inactivity has been linked to the decreased cognitive function and decreased mental health in older adults^24,25^.

This study created a new experimental animal model characterized by high caloric intake and physical inactivity (PI), both classic features of the modern lifestyle. We investigated whether olive leaf extract intake could prevent obesity, cognitive decline, and depression in this model. Besides, we investigated whether olive leaf extract intake could enhance exercise endurance capacity.

## Results

### Olive leaf extract enhances lipolysis in adipose tissue and prevents obesity

**Figure 1:**
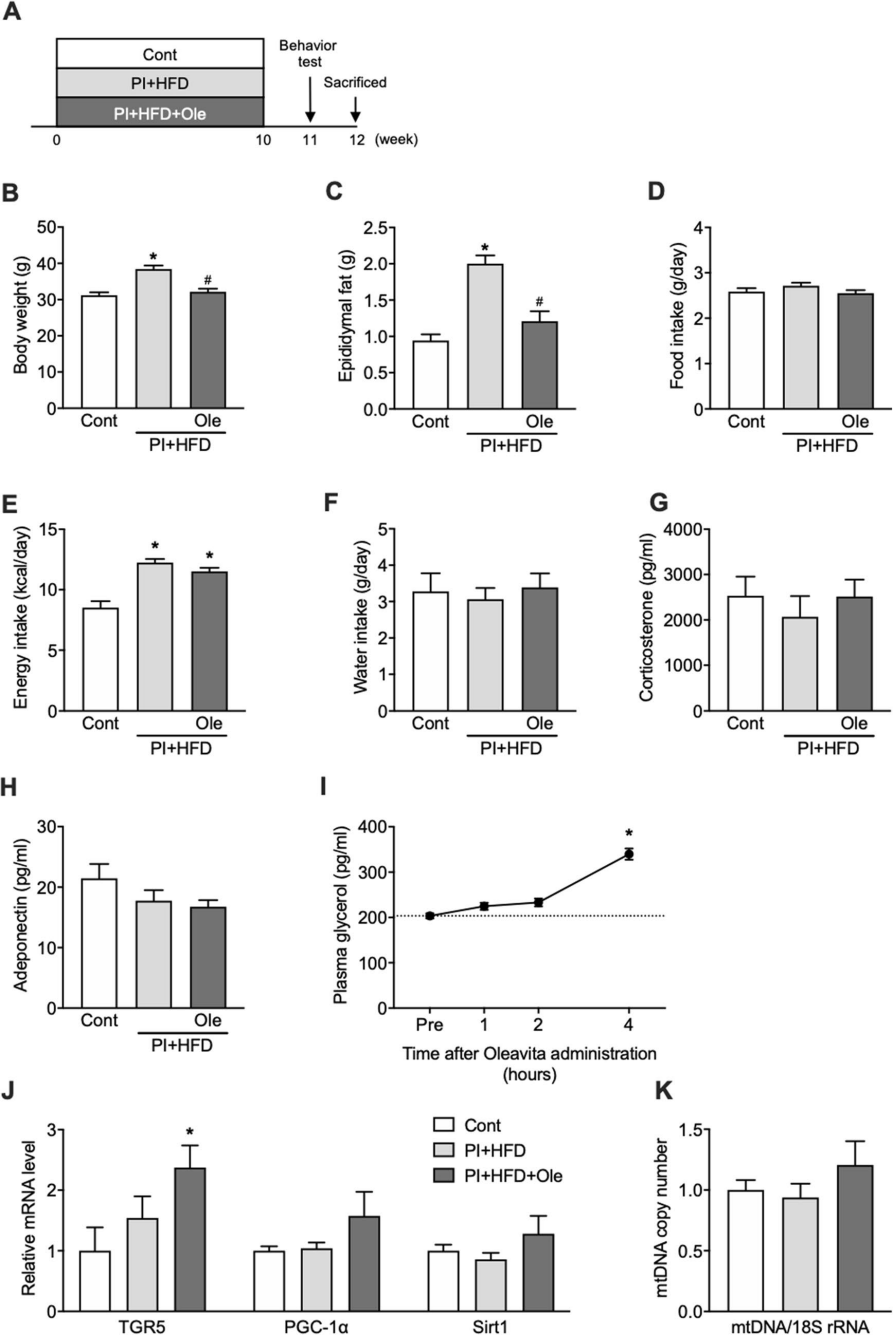
Olive leaf extract prevented gains in body weight and epididymal fat tissue. (**A**) Experimental protocol, (**B**) body weight, (**C**) epididymal adipose fat (EDF) mass, (**D**) food intake, (**E**) energy intake, (**F**) water intake, (**G**) plasma corticosterone concentration, (**H**) plasma adiponectin concentration, (**I**) plasma glycerol concentration after oral administration of olive leaf extract, (**J**) mRNA expression levels of *TGR5*, *PGC-1α*, and *Sirt1* in the EDF by real-time polymerase chain reaction, (**K**) mtDNA copy number in EDF. PI + HFD: physical inactivity (PI) + high-fat diet (HFD), PI + HFD + Ole: PI + HFD + Olive leaf extract. Data are expressed as mean ± SEM. *p < 0.05 vs control, ^#^p < 0.05 vs PI + HFD, as determined by one-way ANOVA.

Rearing mice in physically limiting divided cages for 15 weeks significantly increased body weight and epididymal fat (EDF) mass in PI + HFD mice compared with control mice C (p < 0.0001) (Fig. 1B,C), with no significant differences between control and PI + HFD + Ole mice. Food and water intake did not significantly differ between these three groups (Fig. 1D,F), whereas calorie intake was significantly higher in PI + HFD and PI + HFD + Ole mice than the control mice (p < 0.0001) (Fig. 1E). There were no significant differences in plasma corticosterone and adiponectin levels within these three mice groups (Fig. 1G,H). In another experiment, oral administration of olive leaf extract to mice significantly increased plasma glycerol concentrations, an indicator of lipolysis, 4 h after injection (p < 0.0001) (Fig. 1I). The oleanolic acid in olive leaf extract acts as a TGR5 agonist, enhancing mitochondrial function and increasing lipolysis^9^. Our data showed that *TGR5* mRNA expression in EDF was significantly increased in PI + HFD + Ole mice compared to that in control and PI + HFD mice (p < 0.043)(Fig. 1J). *PGC-1α* and *Sirt1* mRNA expression levels and mitochondrial (mt) DNA copy number tended to be higher in PI + HFD + Ole mice than in the two other groups of mice, without statistical significance (Fig. 1J,K).

### Olive leaf extract enhances antioxidant capacity and citrate synthase activity in the hippocampus and prevents cognitive decline and depressive behavior

**Figure 2:**
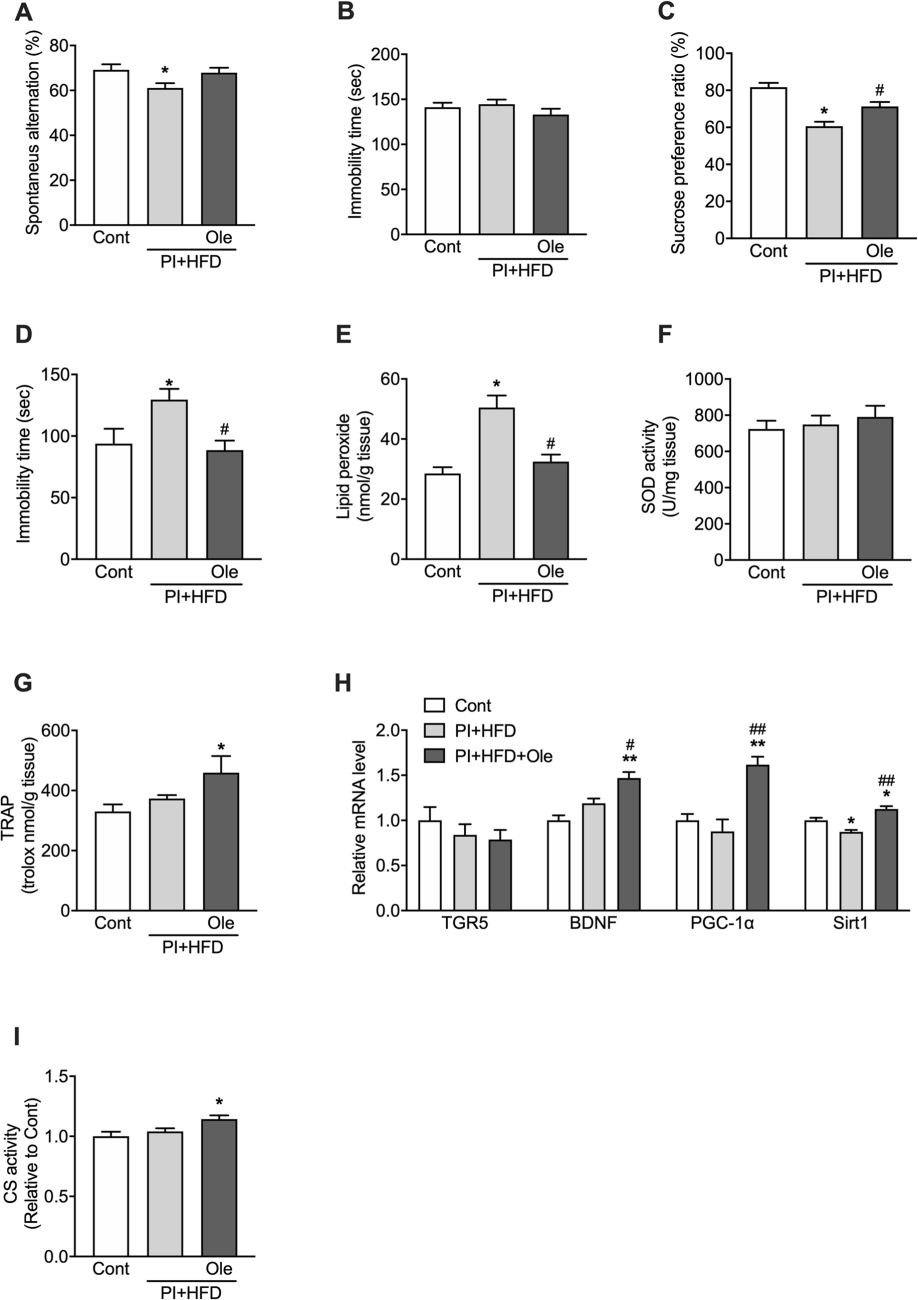
Olive leaf extract (Olea) prevented behavioral changes induced by inactivity and intake of a HFD. (**A**) Accuracy rate in the Y-maze test, (**B**) immobility time in the contextual fear conditioning test, (**C**) sucrose preference ratio in the sucrose preference test, (**D**) immobility time in the forced swim test, (**E**) hippocampal lipid peroxide content, (**F**) hippocampal SOD activity, (**G**) hippocampal TRAP level, (**H**) mRNA expression levels of *TGR5*, *BDNF*, *PGC-1α*, and *Sirt1* in the hippocampus by real-time polymerase chain reaction analysis, and (**I**) CS activity in the hippocampus. Data are expressed as mean ± SEM. *p < 0.05 vs control, ^#^p < 0.05 vs PI + HFD as determined by one-way ANOVA.

Since inactivity and an HFD have been associated with cognitive decline, we performed two behavioral tests to investigate the mice’s cognitive function. In the Y-maze test, which examined working memory, PI + HFD mice showed significantly lower spontaneous alternation than control mice, with PI + HFD + Ole mice showing the same average score as control mice (F_(2,36)_ 3.42; p = 0.040) (Fig. 2A). In the contextual fear-conditioning test (CFCT), which examined long-term memory, there were no significant differences in mice’s immobility times in the three groups (Fig. 2B).

Since an HFD has been shown to cause depressive behaviors^26–28^, we used the sucrose preference test (SPT) and the forced swimming test (FST) to examine this factor. In the SPT, the sucrose preference ratio was significantly lower in PI + HFD mice than in control mice (F_(2,36)_ 19.65; p < 0.0001), with PI + HFD + Ole mice showing no significant decreases (Fig. 2C). Immobility times in the FST was significantly longer in PI + HFD than in control mice (F_(2,31)_ 4.44; p = 0.020), with PI + HFD + Ole mice showing similar immobility times as control mice (Fig. 2D).

Since peroxide accumulation is one cause of the cognitive decline observed during long-term intake of an HFD^6,29^, we measured hippocampal lipid peroxide (LPO) levels in mice. Hippocampal LPO levels were significantly increased in PI + HFD mice compared to that in control mice (F_(2,33)_ 16.75; p < 0.0001), with PI + HFD + Ole mice showing similar LPO levels as those in control mice (Fig. 2E). There were no significant between-group differences in superoxide dismutase (SOD) activity, an enzyme that eliminates reactive oxygen species (Fig. 2F). On the other hand, the total radical trapping antioxidant parameter (TRAP) level measured in the deproteinized fraction of a hippocampal homogenate was significantly higher in PI + HFD + Ole mice than in the other two groups of the mice (F_(2,29)_ 16.75; p < 0.05) (Fig. 2G). Additionally, hippocampal mRNA expression levels of brain-derived neurotrophic factor (*BDNF*), *PGC-1α*, and *Sirt1* were significantly higher in PI + HFD + Ole mice than in the other two groups of mice (p < 0.05) (Fig. 2H), whereas *TGR5* mRNA was not different among the three groups. In contrast, hippocampal citrate synthase (CS) activity was significantly higher in PI + HFD + Ole mice than in the other two groups of mice (F_(2,26)_ 5.18; p < 0.05) (Fig. 2I).

### Oleanolic acid and oleuropein enhance lipolysis and prevent obesity

**Figure 3:**
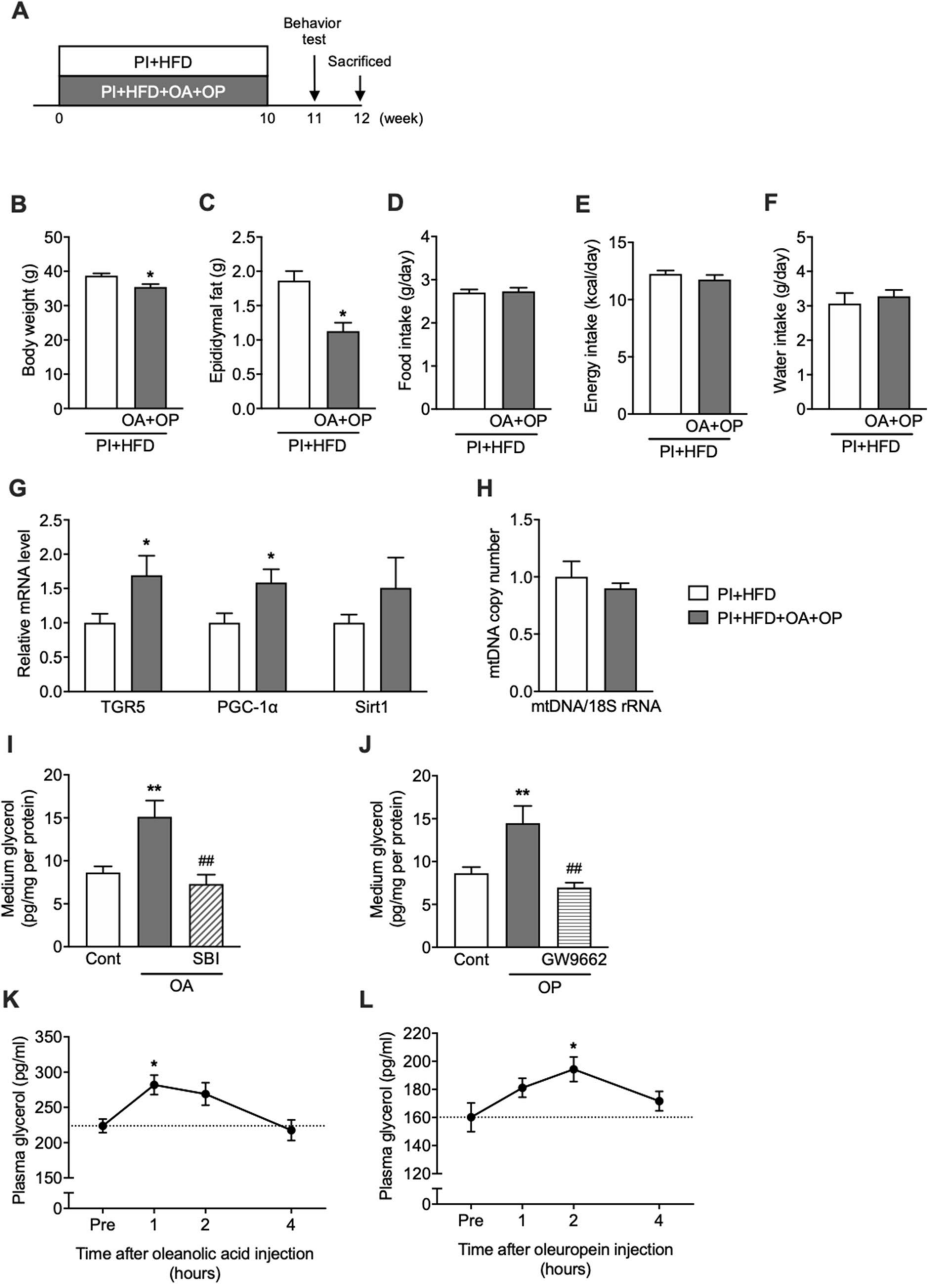
Combined administration of oleanolic acid (OA) and oleuropein (OP) prevented gains in body weight andEDF mass. (**A**) Experimental protocol, (**B**) body weight, (**C**) EDF mass, (**D**) food intake, (**E**) energy intake, (**F**) water intake, (**G**) mRNA expression levels of *TGR5*, *PGC*-1α, and *Sirt1* in EDF, (**H**) mtDNA copy numbers in EDF, (**I**) glycerol released from adipocytes to medium by administration of OA or OA + SBI-155 (TGR5 antagonist), (**J**) glycerol released from adipocytes to medium by administration of OP or OP + GW9662 (PPARγ antagonist), (**K**) plasma glycerol concentration after oral administration of oleanolic acid, or (**L**) oleuropein. Data are expressed as mean ± SEM. *p < 0.05 vs. (physical inactivity) PI + high-fat diet (HFD) as determined by an unpaired, two-tailed *t* test.

Oleanolic acid and oleuropein are major physiologically active components in olive leaf extract. Therefore, we examined whether olive leaf extract’s preventative effects on obesity, cognitive decline, and depression were related to these two substances. Mice receiving oleanolic acid and oleuropein (PI + HFD + OA + OP) showed significantly lower body weights and epididymal fat masses (p < 0.0001) than PI + HFD mice (Fig. 3B,C), with no significant differences in food, energy intake, and water intake (Fig. 3D–F). PI + HFD + OA + OP mice showed significantly higher *TGR5* and *PGC-1α* mRNA expression levels in EDF mass than did PI + HFD mice (p < 0.05) (Fig. 3G), with an increased tendency in *Sirt1* mRNA and no differences in mtDNA levels (Fig. 3H). Experiments with primary cultured adipocytes revealed that the addition of oleanolic acid (F_(2,26)_ 9.73; p < 0.01) or oleuropein (F_(2,20)_ 11.66; p < 0.01) to media increased the amount of glycerol released from adipocytes into the media (p < 0.05) (Fig. 3I,J). Additionally, the addition of TGR5 antagonist SBI-115 or peroxisome proliferator-activated receptor gamma (PPARγ) antagonist GW9662 to the media inhibited oleanolic acid- or oleuropein-induced increase in the amount of glycerol released from adipocytes (Fig. 3I,J). Furthermore, intraperitoneal injections of oleanolic acid or oleuropein in mice raised plasma glycerol concentrations 1 h or 2 h after injection, respectively (p < 0.05) (Fig. 3K,L).

### Oleanolic acid and oleuropein enhance antioxidant capacity and citrate synthase activity in the hippocampus and prevent cognitive decline and depressive behavior

**Figure 4:**
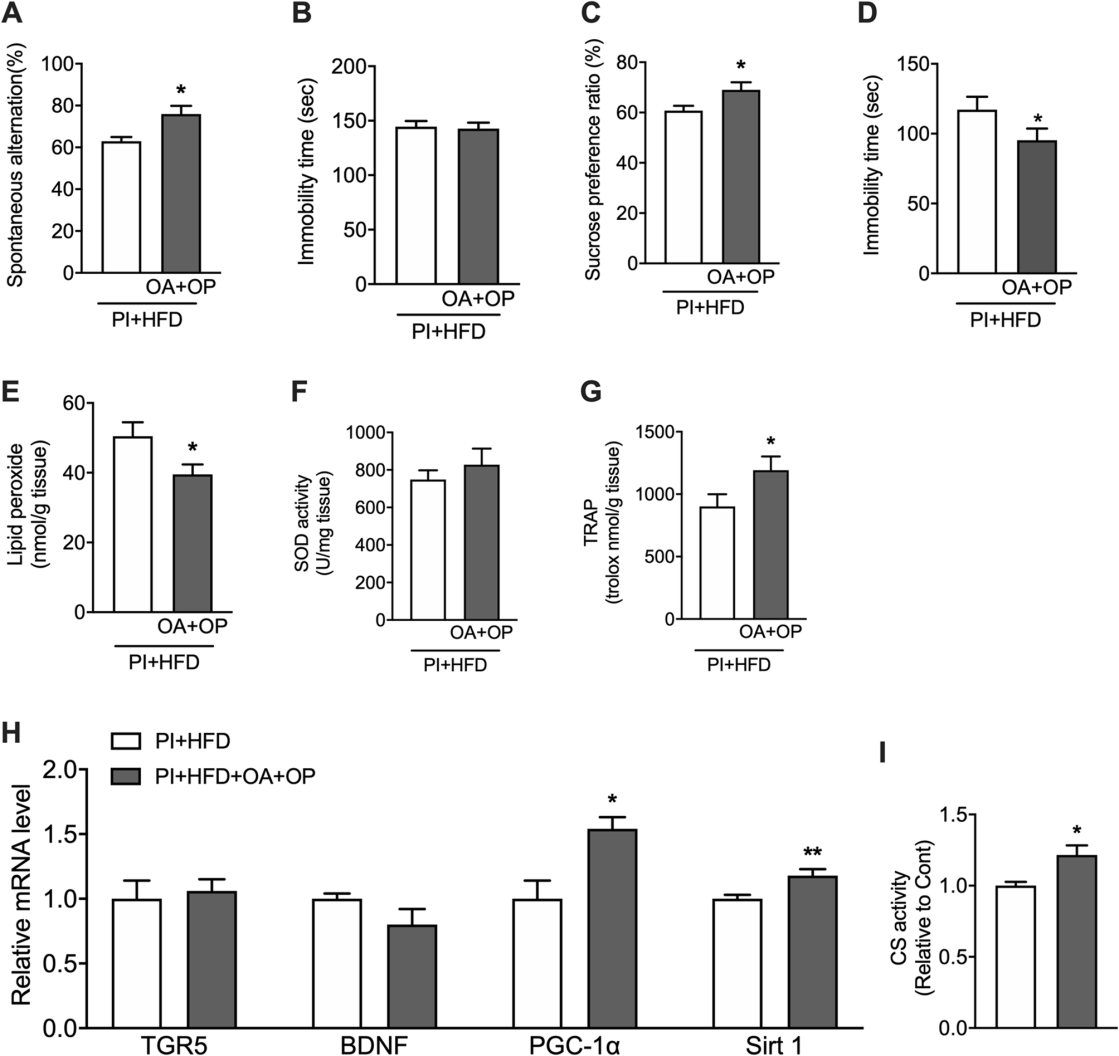
Combined administration of oleanolic acid (OA) and oleuropein (OP) prevented behavioral changes induced by inactivity and a high-fat diet (HFD). (**A**) Accuracy rate in the Y-maze test, (**B**) immobility time in the contextual fear conditioning test, (**C**) sucrose preference ratio in the sucrose preference test, (**D**) immobility time in the forced swim test, (**E**) hippocampal lipid peroxide content, (**F**) hippocampal SOD activity, (**G**) hippocampal TRAP level, (**H**) mRNA expression levels of *TGR5*, *BDNF*, *PGC-1α*, and *Sirt1* in the hippocampus by real-time polymerase chain reaction, (**I**) CS activity. *p < 0.05 vs. PI + HFD as determined by unpaired, two-tailed *t* tests.

In behavioral tests, PI + HFD + OA + OP mice showed significantly higher spontaneous alternation in the Y-maze test than PI + HFD mice (p < 0.01) (Fig. 4A); however, in the CFCT, both groups of mice showed the same immobility times (Fig. 4B). On the other hand, PI + HFD + OA + OP mice showed significantly higher sucrose preference ratios in the SPT and lower immobility times in the FST than did PI + HFD mice (p < 0.05) (Fig. 4C,D). PI + HFD + OA + OP mice also displayed significantly lower hippocampal LPO levels and significantly higher TRAP levels (p < 0.05), without significant change in hippocampal SOD activity (Fig. 4E–G) than PI + HFD mice. Additionally, hippocampal expression levels of *PGC-1α* and *Sirt1* mRNA were significantly higher in PI + HFD + OA + OP mice than in PI + HFD mice (p < 0.05), without significant change in *TGR5* mRNA (Fig. 4H). In contrast, hippocampal CS activity was significantly higher in PI + HFD + OA + OP mice than in PI + HFD mice (p < 0.01) (Fig. 4I).

### Olive leaf extract enhances mitochondrial mass in soleus muscle and endurance exercise capacity

**Figure 5:**
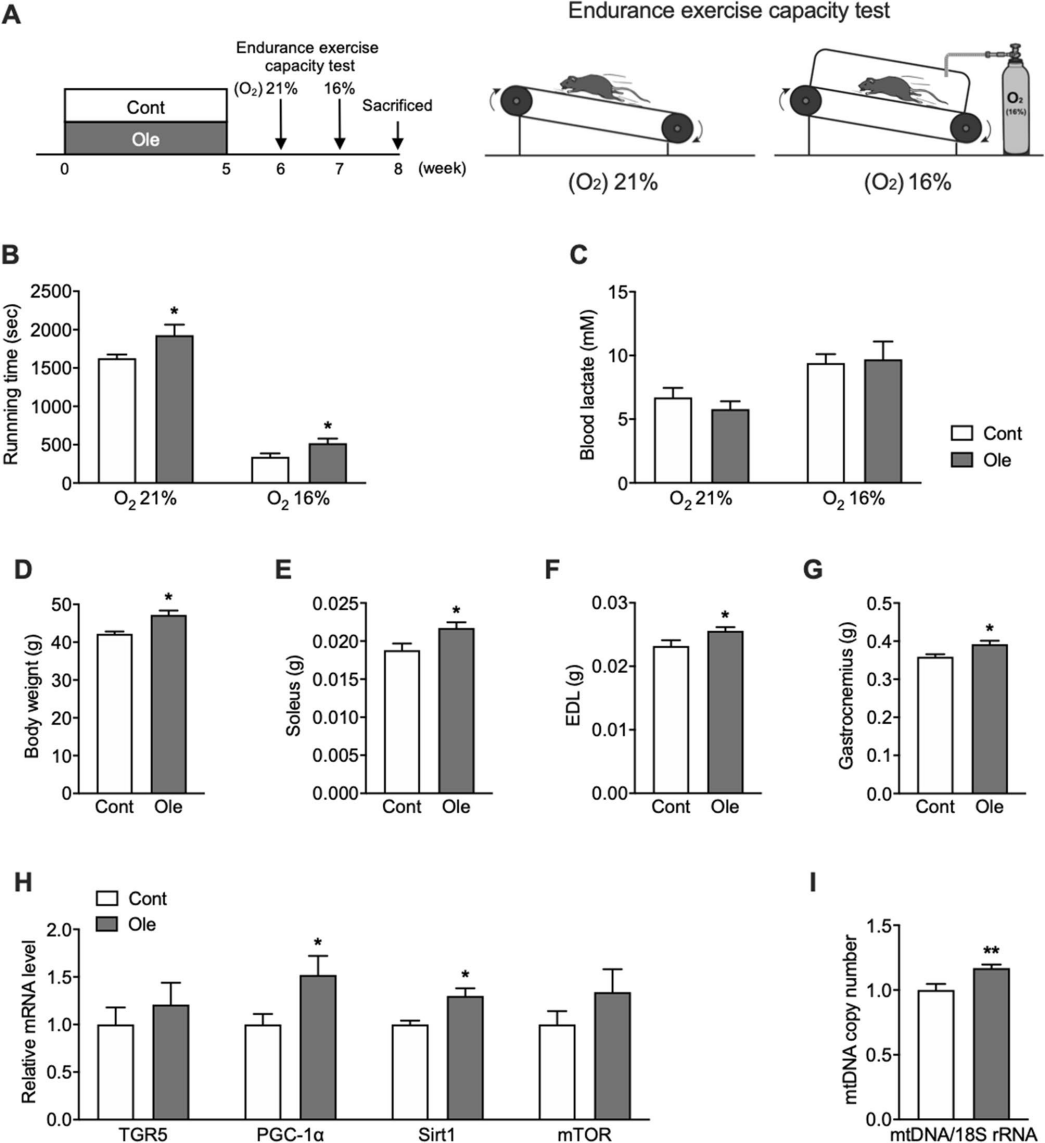
Olive leaf extract improved endurance exercise capacity under atmospheric (O_2_[21%]) and low O_2_ (O_2_[16%]) conditions by enhancing mitochondrial mass in skeletal muscle. (**A**) Experimental protocol; (**B**) running times on a treadmill under standard (atmospheric) and low-O_2_ (16% O_2_) conditions; (**C**) blood lactate concentration just after exercise under standard (atmospheric) and low-O_2_ (16% O_2_) conditions; (**D**) body weight; (**E**–**G**) muscle weights of the soleus, EDL, and gastrocnemius; (**H**) mRNA expression levels of *TGR5*, *PGC-1α*, *Sirt1*, *mTOR* in the soleus muscle; and (**I**) mtDNA copy number in the soleus muscle. *p < 0.05 vs. control as determined by unpaired, two-tailed *t* tests.

Exercise training has been shown to increase mitochondrial biogenesis, contributing to a higher capacity for exercise, especially endurance exercises^30^. Therefore, we examined whether olive leaf extract’s increase in mitochondrial biogenesis could enhance endurance exercise capacity. After 4 weeks of feeding with an olive leaf extract-contained diet or a standard diet, we conducted an endurance running test on a treadmill at a constant speed under atmospheric (oxygen, 21%) or hypoxic (oxygen, 16%) conditions. Exercise duration was markedly decreased in both groups of mice when tested under hypoxic conditions; however, mice fed olive leaf extract showed a significantly longer exercise time than control mice under both atmospheric (p < 0.05) and hypoxic conditions (p < 0.01) (Fig. 5B). Blood lactate levels measured at the end of exercise were significantly higher under hypoxic versus atmospheric conditions; however, there were no significant differences between these two groups of mice’s blood lactate levels under each of these conditions (Fig. 5C). Soleus, extensor digitorum longus (EDL), and gastrocnemius muscle weights were significantly higher in mice fed olive leaf extract than in control mice (p < 0.05), even though body weight was also significantly higher (p < 0.01) (Fig. 5D–G) in mice fed olive leaf extract. Also, *TGR5* and mammalian target of rapamycin (*mTOR*) mRNA levels in the soleus muscle tended to be higher in mice fed olive leaf extract; however, this trend was not significant (Fig. 5H). Furthermore, the levels of *PGC-1α* mRNA (p < 0.05), *Sirt1* mRNA (p < 0.05), and mtDNA copy number (p < 0.01) in the soleus muscle were significantly higher in mice fed olive leaf extract than in control mice (Fig. 5H,I).

## Discussion

In the present study, we investigated olive leaf extract’s effects on obesity, cognitive decline, depression, and endurance exercise capacity. Our results indicated that olive leaf extract administration prevented obesity, cognitive decline, and depression in an inactive mouse model fed an HFD. Oleanolic acid, a component of olive leaf extract, is an agonist of TGR5, a ubiquitous receptor that recognizes bile acids as ligands and increases intracellular cAMP levels^10^. These increased cAMP levels have been shown to cause a rise in β-oxidation and energy production, resulting in fat reduction and anti-obesity effects^10^. Similar to our findings, previous studies have also demonstrated that oleanolic acid administration resulted in decreased fat mass and body weight in mice fed an HFD^11^.

Another main component of olive leaf extract is oleuropein. Oleuropein has been shown to have potent antioxidant and anti-inflammatory actions^8^. Though no previous study has reported that oleuropein has a lipolytic effect, we examined the lipolytic effects of oleuropein and oleanolic acid in vitro and in vivo. The addition of oleanolic acid or oleuropein to culture media increased glycerol levels released from adipocytes (Fig. 3I,J), and intraperitoneal injections of oleanolic acid or oleuropein raised plasma glycerol levels in mice (Fig. 3K,L). Oral administration of olive leaf extract also increased plasma glycerol concentrations 4 h after administration (Fig. 1I). Together, these results suggest that oleanolic acid can increase lipolysis in vitro and in vivo, lending further support to the theory that the oleanolic acid in olive leaf extract can prevent HFD-induced obesity. These results were also in agreement with previous studies showing that administration of nomilin and obacunone^31^, substances found in citrus plants that also have TGR5 agonistic activity, also decrease adipose tissue weight in mice fed an HFD. Additionally, our data showed that oleuropein in the olive leaf extract increases lipolysis in adipose tissue similarly to oleanolic acid, contributing to the anti-obesity effect derived from taking olive leaf extract.

One of the most notable results of this study was that olive leaf extract administration prevented the working memory declines observed after intake of an HFD and physical inactivity. It has been known that chronic stress, including isolation stress, results in cognitive decline and depressive behaviors along with increased plasma corticosterone concentration^32^. However, in the present study, the plasma corticosterone concentration at the point of the dissection did not significantly differ between the control mice and the mice reared inactively (Fig. 1G). When the mice were reared in the divided cage, it could be that there was some stress from the viewpoint of blocking mice’s physical contact with each other, but each mouse was able to see other mice next to it. Therefore, we expected that the isolation stress derived by being reared in the divided cage could slightly contribute to the cognitive decline and depressive behaviors observed in the present study. In contrast, physical inactivity and excess calorie intake could make a considerable contribution.

Decreased mitochondrial function in neurons is the leading cause of cognitive function deterioration, including decreases in working memory^16^. In this study, both the increase of *PGC-1α* and *Sirt1* mRNA expression and CS activity in the hippocampus of the mice fed olive leaf extract or co-fed oleanolic acid and oleuropein was observed (Figs. 2H,I, 4H,I), suggesting that the increase in the mitochondrial function, potentially by increasing mitochondrial biogenesis via *PGC-1α*, would have been contributed to the maintenance of working memory (Figs. 2H, 4H). At this time, adipose tissue *TGR5* mRNA was increased by both olive leaf extract or coadministration of oleanolic acid and oleuropein (Figs. 1J, 3G), which is in agreement with previous studies showing that cholic acid, an agonist for *TGR5*, increases *TGR5* mRNA in adipose tissue^33^. On the other hand, hippocampal *TGR5* mRNA was not increased (Figs. 2H, 4H). We could not elucidate why the hippocampal CS activity of the mice fed olive leaf extract or co-fed oleanolic acid and oleuropein was increased without the enhancement of hippocampal *TGR5* mRNA. The elucidation of this issue will have to be performed in future studies.

Moreover, hippocampal *BDNF* mRNA expression levels were also increased in the mice administered olive leaf extract, which was consistent with previous studies showing that increased PGC-1α caused induction of BDNF^34^. BDNF is essential for maintaining neuronal function; therefore, this increase in *BDNF* mRNA levels may also help maintain working memory in the mice administered olive leaf extract. However, the mice administered oleanolic acid and oleuropein showed improved working memory without the increased hippocampal *BDNF* mRNA (Fig. 4H). Therefore, we cannot explain why OA + OP administration prevented cognitive decline and depressive behaviors from the perspective of increased *BDNF* mRNA expression. However, simultaneous administration of oleanolic acid and oleuropein increased the antioxidant capacity and citrate synthetase activity similarly to that of olive leaf extract administration. Therefore, these results suggest that the improvement in antioxidant capacity or mitochondrial function could be critical to preventing cognitive decline and depressive behaviors.

Furthermore, intake of an HFD has also been shown to induce higher levels of reactive oxygen species (ROS) in the brain and promote cognitive impairment^29,35^. In this study, administration of olive leaf extract or a combination of oleanolic acid/oleuropein reduced HFD-induced increases in hippocampal LPO levels (Figs. 2E, 4E). While there were no significant changes in hippocampal SOD activity (Figs. 2F, 4F), TRAP, which measures the antioxidant capacity of the deproteinated fraction of hippocampal cytosol, was significantly increased in mice fed olive leaf extract or oleanolic acid/oleuropein (Figs. 2G, 4G). These results suggest that olive leaf extract may have antioxidant properties not mediated by SOD. The oleuropein in olive leaf extract has been shown to have strong antioxidant properties ^8^; therefore, oleuropein may have suppressed the increases in reactive oxygen species that are associated with an HFD, contributing to the maintenance of working memory.

Another significant result of this study was that olive leaf extract prevented depressive behaviors induced by an HFD. Feeding of an HFD has been shown to cause depressive behaviors in experimental animals^26–28^. Furthermore, obesity is a risk factor for depression in human studies^36,37^, and reducing obesity has been reported to improve depression^38^. Neuroinflammation and oxidative stress have been postulated to underlie the depression associated with the intake of an HFD^39^. Oleuropein, a component of olive leaf extract, has been shown to have potent antioxidant and anti-inflammatory actions^8^. Our study suggests that oleuropein’s presence in olive leaf extract may prevent depressive behaviors by enhancing antioxidant capacity (Figs. 2G, 4G) and anti-inflammatory actions. Our results were consistent with a previous finding that astaxanthin, an antioxidant with similarities to oleuropein, could inhibit depressive behaviors in mice fed an HFD^27^.

Furthermore, another significant result of this study was that continuous administration of olive leaf extract significantly improved the endurance exercise capacity of mice, and we believe that the oleanolic acid contained in olive leaf extract may have induced this effect. As mentioned previously, oleanolic acid is an agonist of TGR5, and activation of TGR5 increases intracellular cAMP levels, which leads to an increase in PGC-1α followed by mitochondrial biosynthesis^14^. In this study, significant increases in *PGC-1α* and *Sirt1* mRNA expression levels and mtDNA copy number in the soleus muscle of olive leaf extract-treated mice were observed (Fig. 5H,I). These results indicated that olive leaf extract increased mitochondrial mass in the soleus muscle, which potentially improved the endurance exercise capacity observed in these mice (Fig. 5B). Exercise training has been shown to enhance mitochondrial biogenesis in skeletal muscle, thereby improving endurance exercise capacity^40^. In our study, percentage improvements in running times induced by olive leaf extract were approximately 10% and 40% under atmospheric and low O_2_ conditions, respectively (Fig. 5B). It was interesting to note that the incremental changes in running times under low O_2_ conditions were higher than under atmospheric conditions. In low O_2_ conditions, less oxygen is taken into the body; therefore, increases in mitochondrial mass are critical for producing ATP under these conditions. Accordingly, olive leaf extract-induced increases in mitochondrial mass may be more effective at increasing exercise capacity under low O_2_ conditions than under atmospheric conditions.

In this study, we investigated the effect of olive leaf extract by focusing on oleanolic acid and oleuropein contained in the olive leaf extract. However, olive leaf extract contains many other components. We did not examine the contribution of other components on the effects of olive leaf extract observed in the present study. Therefore, we need to examine the contribution of other components in future studies. In addition, we did not examine the effects of a combination of olive leaf extract administration and exercise training on endurance exercise capacity. However, in a previous study, exercise training was performed while administering resveratrol, a sirtuin activator, and the observed anti-obesity effects were similar to those induced by olive leaf extract. An increase in endurance exercise capacity due to exercise training was not further enhanced by the combined use of resveratrol^41^. In future studies, we hope to elucidate whether combining exercise training and olive leaf extract ingestion can further improve endurance exercise capacity. It is promising to find that olive leaf extract improved endurance capacity without engagement in regular physical exercise, suggesting that this substance may be useful for people who are unable to exercise sufficiently to maintain physical fitness.

In conclusion, olive leaf extract administration to physically inactive mice fed an HFD suppressed an increase in fat mass and weight and prevented cognitive decline; it mainly prevented a decrease in working memory and delayed the onset of depressive behaviors. The mice administered olive leaf extract showed increased CS activity in the hippocampus and mtDNA copy number in the adipose tissue. These results show that there is a possibility that the administration of olive leaf extract may increase mitochondrial function, which could be related to changes in body composition and behaviors. This possibility needs to be proved by future studies. In another experiment, olive leaf extract increased endurance exercise capacity under atmospheric and low oxygen conditions. Therefore, olive leaf extract may be a powerful tool to help patients maintain physical and mental health by inducing effects similar to exercise.

## Methods

### Ethical approval

Animal use and procedures were in accordance with the National Institute of Health guideline and approved by the Animal Care and Use Committee of Nippon Medical School (approval no. 30-029). We conducted all efforts to minimize animal pain and discomfort. This study was carried out in compliance with the ARRIVE guidelines.

### Animals

We used 120 male C57BL/6J mice (Sankyo Lab Service, Tokyo, Japan) aged 10 weeks, weighing 20-22 g, and kept under controlled conditions of 24 °C and 50% humidity. The mice were fed tap water and food ad libitum, with a standard day-night cycle of 12 h induced by switching room lights on between 8:00 a.m. and 8:00 p.m.

### Olive leaf extract

We used OLEAVITA for the olive leaf extract (Phytodia S.A.S., Illkirch-Graffenstaden, France). OLEAVITA is an ethanol/water extract of olive leaves and has been shown to contain 5% or more of both oleanolic acid and oleuropein^12^.

### Rearing conditions

In this study, we examined whether olive leaf extract administration was effective in preventing cognitive decline and depressive behaviors in physically inactive (PI) mice fed a high-fat diet (HFD). We divided mice into the following three groups:

1. Mice reared in a standard cage and fed a standard diet (control).
2. Mice reared in divided cages and fed an HFD (PI + HDF).
3. Mice reared in divided cages and fed an HFD containing olive leaf extract (PI + HFD + Ole).

The divided cages used in the present study were created to suppress the mice's daily physical activity by subdividing a standard mouse cage into six compartments using plastic boards ^42,43^. We used a Western diet (F2WTD) as an HFD and a standard diet as per a Western diet (F-93G-MC). Both diets were purchased from Oriental Yeast Co. LTD. (Tokyo, Japan). The control mice were fed with a standard diet prepared as a control for the Western diet. The nutrient and calorie content per 100 g diet of the Western and standard diet was as follows. Standard diet (protein, 20.0 g; fat, 7.0 g; carbohydrate 63.0 g; calorie, 415.0 kcal); Western diet (protein, 17.8 g; fat, 20.0 g; carbohydrate 49.0 g; calorie, 450.8 kcal). Other nutrients (vitamins, minerals, etc.) according to weight, were the same among the two diets.

To prepare the standard diet for feeding to the mice, water was added, and then it was mixed, kneaded, and rounded into pasty sections, after which it was cut and dried. For the HFD, we added 200 g of butter to 800 g of a Western diet, mixed it uniformly, and added 80 ml of water to the kneaded diet, after which we mixed it again, then cut it into small sections and dried it. The HFD diet containing olive leaf extract was made by adding 1 g of olive leaf extract per 1000 g of HFD. Mice were fed these diets for 10 weeks and then subjected to the behavioral tests mentioned below (Fig. 1A). For another experiment, mice were reared under two different experimental conditions for 10 weeks, including (1) mice reared in divided cages and fed an HFD (PI + HFD) and (2) mice reared in divided cages and fed an HFD containing oleanolic acid and oleuropein (PI + HFD + OA + OP). The HFD containing oleanolic acid and oleuropein was made by adding 100 mg of both oleanolic acid and oleuropein per 1000 g of HFD. Ten weeks later, these mice were also subjected to behavioral tests followed by dissection (Fig. 3A).

### Behavioral tests

Ten weeks after study initiation, we conducted four behavioral tests, including the FST, SPT, CFCT. For evaluation of depression-like behaviors, the SFT and FST were conducted, according to the methods of Covington et al.^44^ and Porsolt et al.^45^, respectively. For the SFT, mice were accustomed to drinking from two bottles in their home cages for 3 days before the test. The mice were then subjected to 3 days of a free-choice test between water and a 1% sucrose solution. The bottle positioning was changed daily from the left to the right to eliminate any potential effects of positioning on water consumption. Intake of water and sucrose during a 12-h dark cycle was measured by weighing the bottles before and after the test. The sucrose preference ratio was calculated as a percentage as follows: [sucrose consumption/water and sucrose consumption × 100].

For the FST, mice were placed in a water-filled (25ºC) cylinder with a height of 2 cm and a diameter of 15 cm and video-recorded for 6 min. Immobility times were determined using analytical software based on how long mice floated during the last 4 min of the test (Smart Junior ver.1.0.06, Panlab Inc., Spain).

For examining long-term memory, the CFCT was performed according to published protocols with slight modifications^46^. The mice were placed in a test shock box (model MK-450MSQ, Muromachi Kikai CO. LTD, Japan) and were subjected to three electric foot shocks (0.8 mA, 2-s duration, and 2-min interval) 2 min later. Mice were left in the apparatus for 1 min more and then returned to their home cages. Twenty-four hours later, the mice were again placed in the test box and video recorded for 3 min to measure immobility times using the same software.

A Y-maze test was conducted to examine working memory based on the methods of Rubaj et al.^47^ with slight modifications. The Y maze consisted of three equally spaced arms (length: 40 cm, height: 12 cm, width: 3 cm). Mice were placed at the end of one arm and were allowed to traverse within the apparatus freely, while being video-recorded for 8 min. Complete entry was determined when a mouse’s hind paws had entirely entered any arm of the maze. The ‘right’ choice was defined as consecutive entry into three different arms. Spontaneous alternation was calculated as the percentage of correct entries to the total number of entries.

### Dissection and sample collection

After completing all behavioral tests, mice were weighed and sacrificed by decapitation, and blood samples were collected. Brain, epididymal adipose fat, soleus muscle, and EDL muscle were then harvested, and the hippocampus was quickly isolated from the brain. Subsequently, the weights of the soleus, EDL, and epididymal adipose tissue were measured. Blood samples were centrifuged at 1500×*g* for 20 min to separate plasma. Collected tissues and plasma were then frozen by liquid nitrogen and stored at − 80°C until analysis.

### Plasma corticosterone and adiponectin concentration

Plasma corticosterone and adiponectin concentrations were measured using the Corticosterone ELISA Kit (Cayman Chem., USA) and Circulex Mouse Adiponectin ELISA Kit (MBL Life Science, Japan), respectively, according to the user manuals.

### Superoxide dismutase activity, total radical trapping antioxidant parameter, and lipid peroxide levels

Hippocampi were homogenized in 100-mM Tri-HCl buffer (pH 7.4) using a Bioruptor UCD-250 (Cosmo Bio Inc., Japan). The homogenate was centrifuged at 750×*g* for 10 min. A portion of the supernatant was used to analyze superoxide dismutase (SOD) activity using a SOD activity analysis kit according to the supplier’s instructions (Dojin Science Inc., Japan). The remainder of the supernatant was used to collect the cytosolic fraction using a ProteoExtract Subcellular Proteome Extraction Kit (Merck Millipore Co., USA) according to the kit’s manual. Collected cytosolic fractions were filtered using an ultrafree MC filter (Merck Millipore Co., USA, pore size = 3000 A) to remove protein from the cytosolic fraction and were then used to measure the total radical trapping antioxidant parameter (TRAP) according to the methods of Mikami et al.^48^. TRAP level was defined as the level of water-soluble tocopherol Trolox (Aldrich, USA), which is known to trap two radicals per molecule. The rest of the homogenate was then used to measure LPO levels by a lipid hydroperoxide analysis kit (Cayman Chemical, Inc., USA).

### RNA extraction and real-time PCR

The hippocampal and muscular samples of mice were homogenized in TRIzol Reagent (Invitrogen, CA, USA) on ice, and total RNA was extracted according to the manufacturer’s instructions. Total RNA was quantified using absorption at 260 nm and the 260:280 nm ratio in order to assess concentration and purity, respectively. Complementary DNA (cDNA) was synthesized using 1 μg of total RNA in a 20-μL reaction with oligo (dT)12–18 primers (Invitrogen) and the ReverTra Ace cDNA reverse transcription kit (Toyobo, Osaka, Japan) according to the manufacturer’s instructions. Quantitative real-time PCR was performed with the SsoAdvanced Universal SYBR Green Supermix (Bio-Rad) and a CFX Connect real-time PCR system (Bio-Rad) to quantify the mRNA levels of *TGR5*, *BDNF*, *PGC-1α*, *Sirt1* and *mTOR*. Glyceraldehyde-3-phosphate dehydrogenase (*GAPDH*) was used as the endogenous control. The oligonucleotide sequences used for analysis are shown in Table S1. The 2-ΔΔCt method was used to analyze relative mRNA expression values ^49^. Sample analysis for each gene was performed in duplicate.

### Mitochondrial DNA copy number

For quantifying adipose tissue and soleus mtDNA, total DNA was extracted from the adipose tissue and soleus muscle using the phenol–chloroform method. Approximately 20 mg of the adipose tissue or soleus muscle was dissolved in 200 μL of lysate buffer containing 10 mM Tris–HCl (pH 8.0), 150 mM NaCl, 10 mM EDTA (pH 8.0), 0.1% SDS, and 2.5% proteinase K. Next, it was vortexed, incubated at 56 °C for 90 min, and centrifuged at 2,000*g* for 10 min. The supernatant was mixed with an equal volume of phenol/chloroform/isoamyl alcohol, vortexed, and centrifuged at 2,000*g* for 10 min. Then, the supernatant that was obtained was mixed with two volumes of 100% ethanol by slow inversion and centrifuged at 10,000*g* for 10 min. After the supernatant was removed, the precipitate was dissolved in 70% ethanol by slow inversion and centrifuged at 10,000*g* for 10 min. Subsequently, after the supernatant was removed, the precipitate was left for 5 min at room temperature, dissolved in Tris–EDTA (TE) buffer (pH 8.0), and stored at − 20 °C until analysis. DNA concentration and purity were analyzed using absorption at 260 nm and the 260:280 nm absorption ratio, respectively. Quantitative real-time PCR (10 ng DNA) was performed with the SsoAdvanced Universal SYBR Green Supermix. 18S ribosomal RNA (18S rRNA) was used as the nuclear DNA (nDNA) control. The sequences of the oligonucleotide used for mtDNA and nDNA analysis are shown in Table S1. The 2-ΔΔCt method was used to analyze the relative mtDNA to nDNA copy number ratio^49^. Sample analysis for each gene was performed in duplicate.

### Lipolysis in adipocytes in response to oleanolic acid and oleuropein

We examined the lipolytic effects of oleanolic acid and oleuropein on primary cultured adipocytes according to the modified methods of Rodbel^50^. Briefly, the collected adipocytes from the mice fed with the standard diet mentioned above were incubated in an MEM medium containing 10% fetal bovine serum for 3 h and then incubated with 10 μM oleanolic acid or 1 μM oleuropein for 4 h. In another experiment, since oleanolic works via TRG5 and oleuropein dose via PPARγ, one hour before adding oleanolic acid or oleuropein to the medium, adipocytes were added 200 μM SBI-115 (TGR5 antagonist) or 10 μM GW9662 (PPARγ antagonist) to the medium and incubated ​for 4 h. The adipocytes and medium were then collected, and the concentration of free glycerol released from adipocytes into the medium was measured using a glycerol assay kit (Sigma-Aldrich, USA). The amount of released glycerol was calculated as pg per mg of protein, with the amount of adipocyte protein measured using a Pierce 660-nm protein assay reagent (Thermo Scientific, USA).

### Lipolysis in vivo via olive leaf extract, oleanolic acid and oleuropein

We examined the lipolysis effect of olive leaf extract, oleanolic acid, oleuropein in vivo using the mice fed a standard diet and fasted for 16 h. Mice were intraperitoneally administered oleanolic acid (100 mg/kg of body weight) or oleuropein (100 mg/kg of body weight), and then their blood samples were collected from the tail vein at 1, 2, and 4 h after the injection. In another experiment, the mice were orally administered olive leaf extract (2,000 mg/kg of body weight) dissolved in water using a gavage probe, and their blood samples were collected from the tail vein at 1, 2, and 4 h after the injection. We determined the administered dose of oleanolic acid based on the assumption that olive leaf extract contained 5% of in-weight % oleanolic acid. Immediately afterwards, blood was centrifuged, and the plasma was then collected and stored at − 80 °C until analysis. The lipolytic effects of olive leaf extract, oleanolic acid and oleuropein were examined by measuring plasma glycerol levels using a glycerol assay kit.

### Endurance running under atmospheric and low-oxygen conditions

We investigated whether administration of olive leaf extract improved endurance exercise capacity. Male ICR mice aged 8 weeks were divided into two groups: (1) mice fed a standard diet (control) and (2) mice fed a standard diet containing olive leaf extract. Both groups of the mice were reared in the standard cage at four mice per cage. Before starting the feeding experiment, all the mice were subjected to running on the treadmill at the speed of 10 m/min, 5 min per day during a week to habituate them to treadmill running. During the feeding experiment, the mice ran on a treadmill at a treadmill speed of 10 m/min for 10 min two times per week. After 5 weeks of feeding the diet containing olive leaf extract, an endurance exercise test was then performed on the treadmill under atmospheric conditions. One week later, the same endurance exercise test was conducted on a closed treadmill filled with 16% oxygen gas. During both tests, the treadmill speed and inclination angle were 20 m/min and 10°, respectively (Fig. 5A). We measured the time until mice became exhausted. We defined as exhaustion time when mice did not return to the treadmill lane from the resting platform three consecutive times. Immediately after the endurance test, blood samples were collected under anesthesia from the tail vein to measure blood lactate levels. Afterward, the mice were dissected by decapitation, and the soleus, EDL and gastrocnemius muscles were collected and stored at − 80 °C.

### Statistical analysis

All data are expressed as mean ± SE and were analyzed using GraphPad Prism 8 (MDF Co., Ltd, Tokyo, Japan). Group comparisons were performed using a one-way ANOVA followed by Dunnett’s and Tukey’s post hoc tests. Time-course changes were compared between pre-exercise and post-exercise levels using a one-way ANOVA followed by *t* test comparisons. Comparisons of two groups were performed using Student’s *t* test for unpaired data. The differences between groups were considered statistically significant at p < 0.05.

## Supplementary Information

Supplementary Information 1.

## Abbreviations

Ole: Olive leaf extract
cAMP: Cyclic adenosine monophosphate
HFD: High-fat diet
PGC-1α: Peroxisome proliferator-activated receptor-gamma coactivator 1α
CFCT: Contextual fear-conditioning test
SPT: Sucrose preference test, FST, forced swimming test
TRAP: Total radical trapping antioxidant parameter
mTOR: Mammalian target of rapamycin
GAPDH: Glyceraldehyde-3-phosphate dehydrogenase
