# Supplementary material for: Olive leaf extract prevents obesity, cognitive decline, and depression and improves exercise capacity in mice
Source: Sci Rep. 2021 Jun 14;11:12495. doi: 10.1038/s41598-021-90589-6 (PMC8203715; doi:10.1038/s41598-021-90589-6)
Supplement: Supplementary file 1 — Supplementary Information 1. [file 41598_2021_90589_MOESM1_ESM.docx]

**Olive leaf extract prevents obesity, cognitive decline, and depression and improves exercise capacity in mice**

Toshio Mikami^1^, Jimmy Kim^2^, Jonghyuk Park^2^, Hyowong Lee^2^, Pongson Yaicharoen3, Sofya Suidasari^4^, Miki Yokozawa^4^, Ken Yamauchi^4^

1 Department of Health and Sports Science, Nippon Medical School, Tokyo 180-0023, Japan
2 Department of Pharmacology, Nippon Medical School, Tokyo 113-8602, Japan
3 Department of Physiology, Chiang Mai University, Chiang Mai, Thailand

4 Nutrition Act. Co. Ltd., Tokyo 104-0061, Japan

**Table S1. Sequences for the primers used for real-time PCR procedures**

| Enzyme/protein  name | Gene  name | Direction | Primer sequences |
| --- | --- | --- | --- |
| mRNA | | | |
| TGR5 | *Tgr5* | Forward | 5′-TACCACACCAGTAGCCAAT-3′ |
|  |  | Reverse | 5′-TGCTGCTTCCCTAATTCAAG-3′ |
| BDNF | *Bdnf* | Forward | 5′-TGCAGGGGCATAGACAAAAGG-3′ |
|  |  | Reverse | 5′-CTTATGAATCGCCAGCCAATTCTC-3′ |
| PGC-1α | *Pgc-1α* | Forward | 5′-ACCCTGCCATTGTTAAGACC-3′ |
|  |  | Reverse | 5′-CTGCTGCTGTTCCTGTTTTC-3′ |
| Sirtuin1 | *Sirt1* | Forward | 5′-ACTCCTCACTAATGGCTTTCATTC-3′ |
|  |  | Reverse | 5′-GGTGGAGGAATTGTTTCTGGTAAT-3′ |
| mTOR | *mTor* | Forward | 5′-AACAACACAGCTGGGGACGA-3′ |
|  |  | Reverse | 5′-TCTCGGAGCACTTCCATCACA-3′ |
| GAPDH | *Gapdh* | Forward | 5′-CATCACTGCCACCCAGAAGA-3′ |
|  |  | Reverse | 5′-ATGTTCTGGGCAGCC-3′ |
| mtDNA | | | |
| COX I |  | Forward | 5′-TGATTCCCATTATTTTCAGGCTTC-3′ |
|  |  | Reverse | 5′-ACTCCTACGAATATGATGGCGAA-3′ |
| 18S rRNA |  | Forward | 5′-CGCCGCTAGAGGTGAAATTC-3′ |
|  |  | Reverse | 5′-CTTGGCAAATGCTTTCGCTC-3′ |

TGR5, transmembrane G protein-coupled receptor 5; BDNF, brain-derived neurotrophic factor; PGC-1α, peroxisome proliferator-activated receptor gamma coactivator; mTOR, mammalian target of rapamycin; GAPDH, glyceraldehyde-3-phosphate dehydrogenase
